# Fucoidan Independently Enhances Activity in Human Immune Cells and Has a Cytostatic Effect on Prostate Cancer Cells in the Presence of Nivolumab

**DOI:** 10.3390/md20010012

**Published:** 2021-12-22

**Authors:** Ah Young Park, Imane Nafia, Damien N. Stringer, Samuel S. Karpiniec, J. Helen Fitton

**Affiliations:** 1 Marinova Pty Ltd., Cambridge, TAS 7170, Australia; damien.stringer@marinova.com.au (D.N.S.); sam.karpiniec@marinova.com.au (S.S.K.); drfitton@rdadvisor.com (J.H.F.); 2 Explicyte Immuno-Oncology, 33000 Bordeaux, France; i.nafia@explicyte.com; 3 RDadvisor, Hobart, TAS 7006, Australia

**Keywords:** fucoidan, Nivolumab, human peripheral blood mononucleocytes, prostate cancer, PC3

## Abstract

Fucoidan compounds may increase immune activity and are known to have cancer inhibitory effects in vitro and in vivo. In this study, we aimed to investigate the effect of fucoidan compounds on ex vivo human peripheral blood mononuclear cells (PBMCs), and to determine their cancer cell killing activity both solely, and in combination with an immune-checkpoint inhibitor drug, Nivolumab. Proliferation of PBMCs and interferon gamma (IFNγ) release were assessed in the presence of fucoidan compounds extracted from *Fucus vesiculosus*, *Undaria pinnatifida* and *Macrocystis pyrifera*. Total cell numbers and cell killing activity were assessed using a hormone resistant prostate cancer cell line, PC3. All fucoidan compounds activated PBMCs, and increased the effects of Nivolumab. All fucoidan compounds had significant direct cytostatic effects on PC3 cells, reducing cancer cell numbers, and PBMCs exhibited cell killing activity as measured by apoptosis. However, there was no fucoidan mediated increase in the cell killing activity. In conclusion, fucoidan compounds promoted proliferation and activity of PBMCs and added to the effects of Nivolumab. Fucoidan compounds all had a direct cytostatic effect on PC3 cells, as shown through their proliferation reduction, while their killing was not increased.

## 1. Introduction

Fucoidans are a class of brown seaweed derived, fucose rich sulfated polysaccharides that are known to have direct and indirect effects on cancer cells in vitro and in vivo [1,2,3,4]. The efficacy of fucoidan as an anti-cancer agent has been extensively explored, especially in nanoparticle platforms and across a range of cancers including head and neck squamous cell carcinoma [5], murine leukaemia and colorectal cancer [6,7], pancreatic cancer [8,9] and breast cancer [10,11]. 

Fucoidans are known, on the one hand, to be ‘anti-inflammatory’ [2,12]. Fucoidan is classically known as a selectin and scavenger receptor blocking agent [13]. By blocking these cellular adhesion molecules, fucoidan can prevent intrusion of neutrophils into tissue spaces, attenuating inflammatory responses. Conversely, fucoidans may also act as immune adjuvants [14,15], recover immunity in immunosuppressed mice [16] and stimulate chemokines, major histocompatibility complex class I and II, and pro-inflammatory cytokines in PBMCs [17]. 

As a promising immune modulator, fucoidan was further explored in possible cancer therapy models. In a mouse model, fucoidan was able to preserve heart muscle cells from damage brought upon by potent antitumor drug doxorubicin [18]. Specificity of fucoidan to P-selectin was applied to target the drug-resistant cancer cells that can survive chemotherapy and develop into metastases [19]. 

Cancer immunotherapy using immune-checkpoint inhibitors (ICI) has achieved great success over the past decade [20]. Nivolumab is a programmed cell death 1 (PD-1) antibody that has achieved clinical success in improving recurrence-free survival in a variety of cancers including melanoma [21], hepatocellular carcinoma [22], non-small cell lung cancer [23] and head and neck cancers [24]. 

Currently, there are few published studies on fucoidan in cancer immunotherapy and overall effects are yet to be fully explored. A recent murine melanoma model study by Yang et al. investigated the role of dietary fucoidans in conjunction with the antitumor activities of PD-1 antibodies [25]. Although fucoidan extracts from *Ascophyllum nodosum* and *Fucus vesiculosus* alone were not able to inhibit melanoma cell growth in vitro and in vivo, fucoidan supplementation in mice significantly reduced tumor volume and weight in the presence of PD-1 antibodies. The authors suggested that fucoidan potentiated ICI therapeutic effects by CD8+ T cell activation through binding to the T cell receptor (TCR)/CD3 complex. 

In another murine study, Chiang et al. reported therapeutic effects of fucoidan in a nanomedicine conjugated with anti-PD-L1 and two T-cell activators, anti-CD3 and anti-CD28 for advance combination immunotherapy [26]. Targeted tumor treatment through magnetic navigation of the nanomedicine extended survival time and minimized adverse effects. 

Intranasal administration of fucoidan extracted from *Ecklonia cava* showed a promising synergistic effect on metastatic lung cancer cells in mice with anti-PD-L1 [27]. Fucoidan activated a variety of immune cells including dendritic cells (DCs), natural killer (NK) cells and T cells in the mediastinal lymph node. 

It is important to screen for interactions between therapeutics and compounds such as fucoidan that may be present in complementary medicines. In this research, we focused on the effects of fucoidans from three different species (*Fucus vesiculosus*, *Undaria pinnatifida* and *Macrocystis pyrifera)* alone, and in combination with a T cell activator (anti-CD3), and an ICI (Nivolumab, anti-PD-1) on human PBMCs and a hormone resistant prostate cancer cell line, PC-3.

## 2. Results

### 2.1. In Vitro Study of the Effects of Fucoidans on the Activation and Proliferation of PBMCs

In order to see whether fucoidans could promote or suppress the activation and proliferation of PBMCs, cell confluence and IFNγ level were quantified in the absence and presence of αCD3. 

#### 2.1.1. Fucoidans Activated PBMCs in the Presence of αCD3

**Figure 1:**
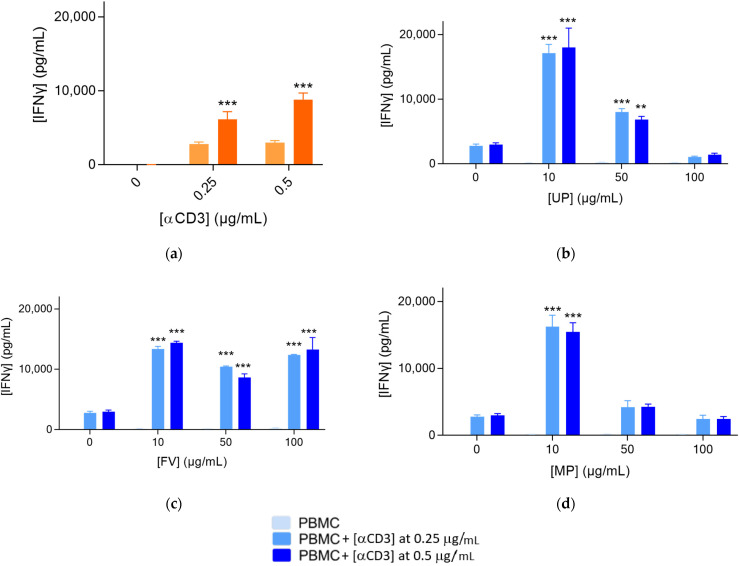
IFNγ levels in culture supernatants 72 h post-culture, measured by HTRF-based technology. (**a**) IFNγ levels released in the supernatants of αCD3-activated PBMC at 0.25 and 0.5 μg/mL cultured for 72 h, in the presence and absence of Nivolumab at 1 μg/mL included as positive control. Inactivated PBMC (without αCD3) was included as a negative control. (**b**–**d**) IFNγ levels released in the supernatants of inactivated and αCD3-activated PBMC at 0.25 and 0.5 μg/mL cultured for 72 h and treated with increasing doses of test compounds UP (**b**), FV (**c**), and MP (**d**). Results are expressed as means ± SEM. ** *p* < 0.01, *** *p* < 0.001 for each condition when compared to the respective control.

The activation of PBMCs was measured through the increased release of IFNγ. As expected, PBMCs cannot be activated alone or by Nivolumab treatment, but in the presence of αCD3, the release of IFNγ significantly increased (Figure 1a). The rise in the concentration of αCD3 did not affect PMBC activation, whereas Nivolumab had a synergistic activity on the IFNγ release. 

The level of IFNγ was slightly increased in the presence of all fucoidans (Figure 1b–d). UP at 50 µg/mL had the highest level of IFNγ (Figure 1b). Under αCD3 stimulation, all fucoidans promoted a strong PBMC activation. The maximal enhancing effect observed was obtained from the lower tested dose at 10 µg/mL and the effect then decreased gradually and reached the baseline level for UP and MP at 100 µg/mL (Figure 1b,d). For FV, PBMC response remained strongly enhanced regardless of the dose. 

#### 2.1.2. Fucoidans Optimise PBMC Clusterisation at Lower Concentrations

**Figure 2:**
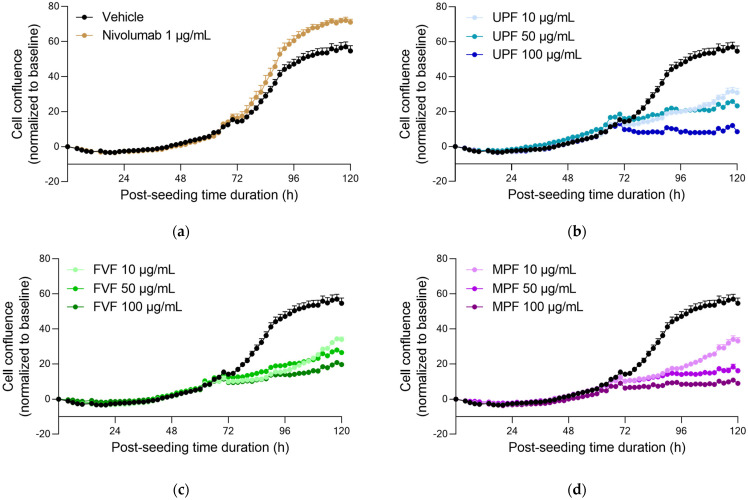
Real-time live cell monitoring of activated PBMCs, under treatment with fucoidans. PBMCs were seeded in the presence of αCD3 (0.50 μg/mL), and treated with increasing doses of Nivolumab, as positive control (*p* < 0.05) (**a**), UP (*p* < 0.001) (**b**), FV (*p* < 0.001) (**c**), and MP (*p* < 0.001) (**d**). Cell confluence was monitored and quantified—as a surrogate of cell proliferation, over a period of ~5 days. Data were normalized and corrected to the baseline and are expressed as means ± SEM.

As expected, proliferation of PBMCs was not observed in the absence of αCD3 (Supplementary Figure S1). Nivolumab treatment induced an optimising effect on cell proliferation, measured by means of cell confluence (Figure 2a). In the presence of αCD3, only a slight rise in kinetics of live cell confluence was observed with fucoidan treatments (Figure 2b–d), which did not directly support a significant increase in the IFNγ release. Fucoidans were shown to optimise PBMC clusterisation at least at the lowest doses (Supplementary Figures S2 and S3), probably due to the compounds’ effects through immune cell activation, rather than through immune cell proliferation promotion. Varying the αCD3 concentration had no effect (Supplementary Figure S4).

### 2.2. Fucoidans Activate PBMCs at Low Concentrations in the Presence of Prostate Cancer Cells

To examine whether fucoidans can activate PBMCs and support the killing of prostate cancer cells, we looked at changes in the IFNγ released level for the immune/tumor co-culture. Since fucoidans at 50 µg/mL or lower concentrations achieved the greatest levels of PBMC activation, we focused on fucoidans in the range of 0 to 50 µg/mL for this study. Hence, we examined IFNγ release in the presence of the PC3 cells at a lower dose of αCD3, while also considering the effect of Nivolumab. 

**Figure 3:**
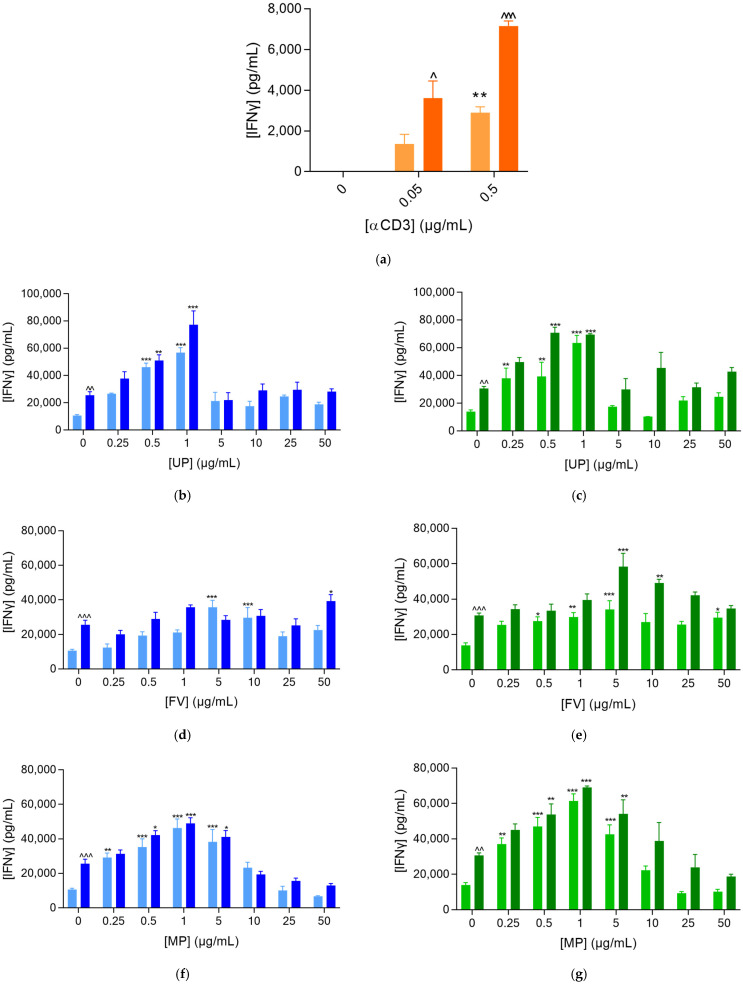

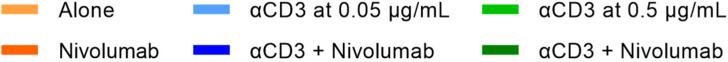
IFNγ levels in culture supernatants 72 h post-initiation of the immune/tumor co-culture, measured by HTRF-based technology on TECAN Spark microplate reader. IFNγ levels released in the supernatants of αCD3-activated PBMC at 0.05 (**b**,**d**,**f**) and 0.5 μg/mL (**c**,**e**,**g**) cultured for 72 h either alone (**a**), or with PC3 tumor cells (10:1 = PBMC:PC3 ratio), in the presence and absence of Nivolumab at 1 μg/mL), and in the presence and absence of UP (**b**,**c**), FV (**d**,**e**), and MP (**f**,**g**). Results are expressed as means ± SEM. * *p* < 0.01, ** *p* < 0.001 and *** *p* < 0.0001 for each condition when compared to the respective control. ^ *p* < 0.01, ^^ *p* < 0.001 and ^^^ *p* < 0.0001 for each Nivolumab-treated condition when compared to the respective Nivolumab-untreated control.

As seen before (Figure 1), the release of IFNγ was promoted significantly—approximately 10-fold—by fucoidan (Figure 3). All fucoidan extracts dose-dependently increased the released levels of IFNγ in the co-culture supernatants, up to 1 µg/mL for UP and MP and up to 5 µg/mL for FV (Figure 3b–g). At higher concentrations, they rather displayed a bell curve effect—the higher the concentration, the lower the optimization of PBMC activation. Moreover, these dose-dependent effects were also independent of the αCD3 doses and were further optimized in the presence of Nivolumab. Therefore, in co-cultures for the immune cell mediated tumor killing assay, PBMC exposure to PC3 tumor cells enhanced αCD3-mediated immune cell activation with respect to supernatants from PBMCs cultured alone. 

### 2.3. Fucoidans Alone Decreased PC3 Tumor Cell Proliferation but Did Not Induce Apoptosis in the Absence of PBMCs

To study the efficacy of fucoidans on PC3 tumor cells, we first looked at the proliferation of PBMCs in the presence and absence of αCD3 at either 0.05 or 0.5 µg/mL and with or without Nivolumab at 1 µg/mL (Supplementary Figure S5) as a control. Proliferation of PBMCs was at the highest level in the presence of αCD3 at 0.5 µg/mL and Nivolumab. Therefore, activation of PBMCs alone was supported by the live cell imaging curves that demonstrated the kinetic proliferation of activated PMBCs and its optimization upon addition of Nivolumab. 

**Figure 4:**
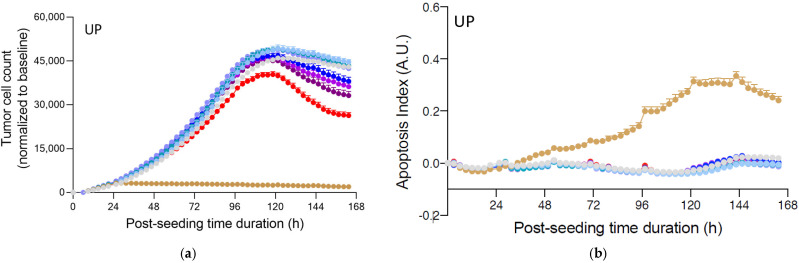

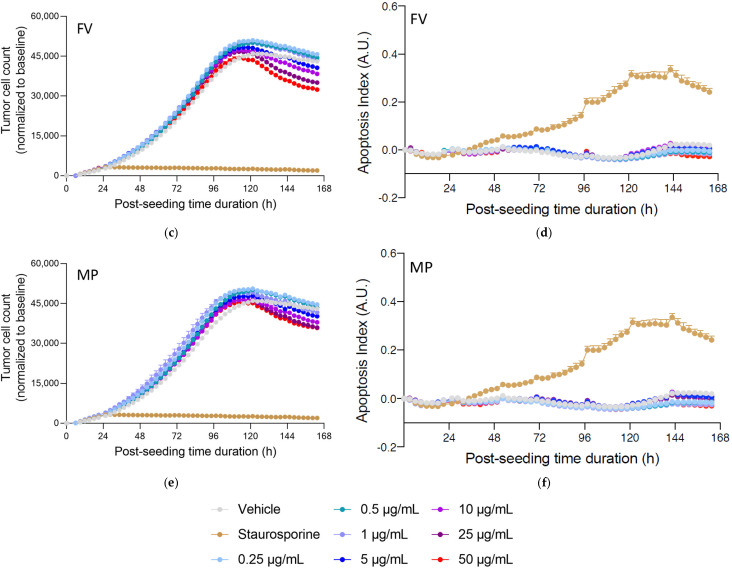
Real-time live cell monitoring of PC3 tumor cell cultures under treatment with fucoidan. PC3 tumor cells were seeded and 24 h later treated with increasing doses of—UP (**a**,**b**), FV (**c**,**d**), and MP (**e**,**f**). Tumor cell count (**a**,**c**,**e**) and apoptosis (**b**,**d**,**f**) were monitored and quantified over a period of ~5 days, by means of NucRed probe expression and caspase 3/7 fluorescent probe, respectively. Apoptosis is represented as an index evaluated with respect to the apoptosis events and cell number in each condition. Data were normalized and corrected to the baseline and are expressed as means ± SEM (*p* < 0.001).

We were interested to see whether fucoidans had any effect of the PC3 cells alone. As expected, treatment of PC3 tumour cells with staurosporine at 10 µM, a known anti-cancer therapeutic, led to a decrease in tumour cell count and a concomitant increase in apoptosis (Figure 4). Under the same conditions, treatment with increasing concentrations of fucoidan extracts dose-dependently induced a moderate and late decrease of tumour cell proliferation, observed starting from 96 h under the three highest concentrations (Figure 4a,c,e). Interestingly, however, no apoptosis was observed to be induced upon treatments (Figure 4b,d,f), thereby suggesting a potential cytostatic effect of the fucoidans. Cell cycle arrest has previously been noted for UP [28]. 

### 2.4. Fucoidans with Activated PBMCs Stopped Tumor Cell Proliferation but Not through Apoptosis

**Figure 5:**
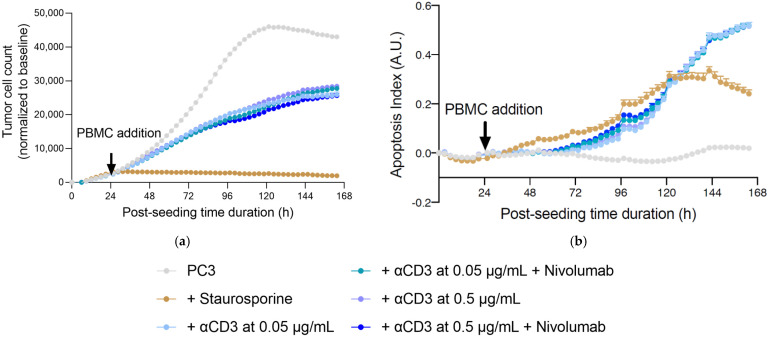
Real-time, live-cell monitoring of prostate PC3 tumor cell killing mediated by activated PBMCs in untreated and Nivolumab-treated conditions. Prostate PC3 tumor cells were seeded and 24 h later were co-cultured with PBMCs, in the presence of αCD3 at 0.05 or 0.5 µg/mL, in the presence and absence of Nivolumab at 1 µg/mL. Tumor cell count (**a**) and apoptosis (**b**) were monitored and quantified over a period of ~5 days, by means of NucRed probe expression and caspase 3/7 fluorescent probes, respectively, as surrogate measures of immune cell killing activity towards tumor cells. Apoptosis is represented as an index evaluated with respect to the apoptosis events and cell number in each condition. Data were normalized and corrected to the baseline and are expressed as means ± SEM (*p* < 0.05).

In co-cultures for the immune cell mediated tumor killing assay, PBMC exposure to PC3 tumor cells led to a decrease in tumor cell count and increase in apoptosis of the prostate PC3 tumor cells (Figure 5).

**Figure 6:**
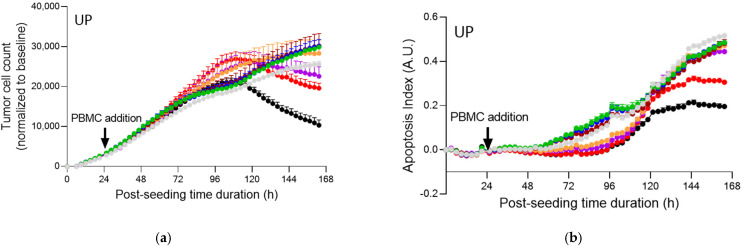

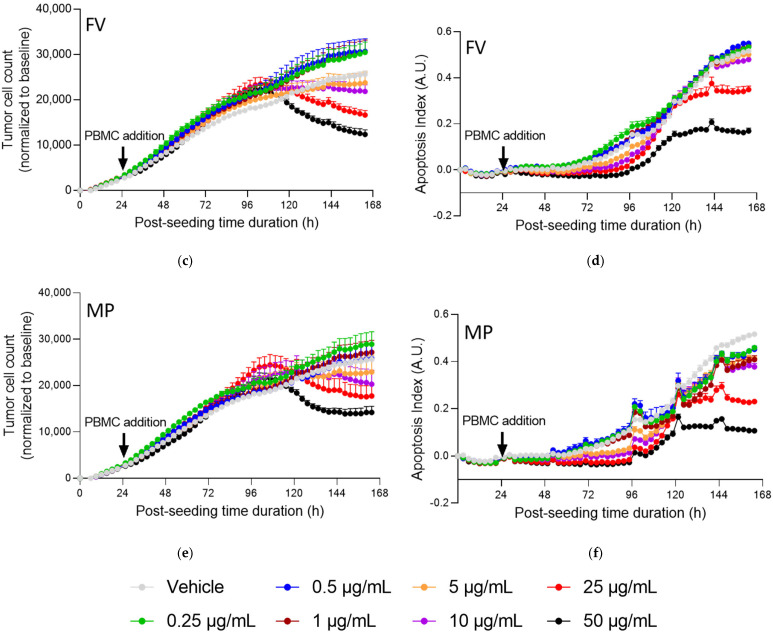
Real-time live cell monitoring of prostate PC3 tumor cell killing mediated by activated PBMCs, under treatment with test compounds. Prostate PC3 tumor cells were seeded and 24 h later were co-cultured with PBMCs, in the presence of αCD3 (0.5 µg/mL), and in the presence of Nivolumab (1 µg/mL), and treated with increasing doses of each of test compounds—UP (**a**,**b**), FV (**c**,**d**), and MP (**e**,**f**). Tumor cell count (**a**,**c**,**e**) and apoptosis (**b**,**d**,**f**) were monitored and quantified over a period of ~5 days, by means of a NucRed probe expression and caspase 3/7 fluorescent probe, respectively, as surrogate measures of immune cell killing activity towards tumor cells. Apoptosis is represented as an index evaluated with respect to the apoptosis events and cell number in each condition. Data were normalized and corrected to the baseline and are expressed as means ± SEM (*p* < 0.001).

Although all fucoidans led to a significantly higher release of IFNγ in PBMCs, these activating effects did not translate into an increase in tumor cell apoptosis and reduction of tumor cell proliferation, at least under the first three tested concentrations (Figure 6a,c,e). However, at higher concentrations, a reduction in tumor cell counts was apparent. 

## 3. Discussion

Prostate cancer is the most diagnosed cancer and is the third leading cause of death in men in the US [29]. Only two checkpoint inhibitors have been approved by the FDA for prostate cancer, and are for a specific metastatic prostate cancer [30]. A small number of clinical trials show promising outcomes with checkpoint inhibitors alone or in combination [31,32]. Clinical studies in Japan showed that *Cladosiphon okamuranus* fucoidan activated NK cells in male cancer survivors [33] and in a normal healthy population group [34]. 

In this study, we investigated effectiveness of fucoidans—available in dietary supplements—in conjunction with anti-PD-1 drug, Nivolumab, on a hormone resistant prostate cancer cell line PC3. 

Although the structure and composition of fucoidan differs between species and extraction procedure, it generally acts as an anti-cancer agent in vitro and in vivo [1,35,36,37,38,39]. Cancer, characterized by abnormal cell growth in an uncontrolled manner, is a multifaceted disease. Fucoidans are thought to act both directly and indirectly. Apoptosis, anti-proliferation and cell cycle arrest [28] are common mechanisms, although there are exceptions where cancer cells are unaffected [40]. The data here for PC3 cells confirm previous data indicating cell cycle arrest in colon cancer cell lines [28]. 

It has been suggested that fucoidans have the potential to inhibit cancer cells indirectly by activating immune cells that suppress tumor growth, although in the data shown here, this does not appear to be the case for PBMCs and PC3 cells. Literature reports show that fucoidan enhances the immune responses of NK cells, T cells, macrophages, DCs and delayed human neutrophil apoptosis [12,41,42,43]. We demonstrated here enhanced IFNγ responses, but these did not correspond to increased cell killing. 

Comparative in vitro studies relying on immune modulation effects of different species of fucoidans suggested that fucoidans from *M. pyrifera* [42] and *Ecklonia cava* [17] were the best candidates for immune priming. In particular, *F. vesiculosus* fucoidan was found to activate human peripheral blood DCs and stimulate antigen-specific T cell immune responses [42,44]. DCs are one of the most important immune cells that bridge innate and adaptive immune systems and also activate T cells. Although usually scarce in the tumor microenvironment, activated DCs can contribute to successful cancer immunotherapies [45,46]. 

In the research described here, UP, FV and MP from similar extraction procedures were shown to activate PBMCs and also synergize activation with anti-CD3 but also particularly with the immune checkpoint inhibitor antibody- anti-PD-1. Interestingly, UP and MP showed bell-shaped dose-response curves, with maximal PBMCs activation at a very low concentration of 1 µg/mL in the presence of the activators. Decrease in IFNγ production at higher concentration of UP and MP is probably due to the cytotoxic effect on PBMCs. This bell-shaped response was somewhat dampened with FV. The reason for the difference in dose-response is not clear, but we speculate that natural polyphenols present in the extract may be in part responsible. There are a number of studies that support the efficacy of natural polyphenols in immune modulation [47]. 

The increase in proliferation of PBMCs by cell confluence was not quantified clearly in the presence of UP, FV and MP due to PBMC clustering. Clustering of PBMCs activated through anti-CD3/IL2 was observed by other researchers in the presence of mebendazole [48]. During an immune response, activated cells of the immune system such as T-cells undergo rapid expansion and many interactions also occur between activated immune cells which may lead to clustering. PBMC clustering for UP and MP was optimized at the lowest tested concentration of 10 µg/mL, and this corresponds well to the maximum released level of IFNγ observed at that concentration.

Remarkably, this increased PBMC activation by fucoidans did not lead to an increase in apoptosis of PC3 cells. Instead, PC3 tumor cell count decreased in a dose-dependent manner via cytostatic effects. Global effects of fucoidan on diverse eukaryotic cellular processes have confirmed effects on cell cycle regulation, DNA damage repair-related pathways and shown induced DNA damage and G1-arrest in colon cancer cells [28]. Importantly, these effects were not observed in primary human fibroblasts. Nevertheless, the exact role of fucoidan interactions in mediating cytostatic effects on prostate cancer cells remains to be further investigated. 

In three recent mice studies with anti-PD-L1, synergistic activity of fucoidans from *Fucus vesiculosus* [25,26], *Ecklonia cava* [27] and *Ascophyllum nodosum* [25] was observed on immune activation. Different types of tumors were investigated in these studies including CT26 colon cancer, 4T1 breast cancer and B16 melanoma. Although fucoidan was not very effective alone, in a combination therapy with the checkpoint inhibitor, the tumor growth was noticeably halted and survival time of mice increased. 

Interestingly, the three studies explored different methods of fucoidan administration including oral [25], intravenous [26] and intranasal [27]. 

Fucoidan extracts have attained regulatory approvals in a number of global jurisdictions for use in foods and dietary supplements. Although the oral bioavailability of fucoidan is generally low due to its size [49,50], systemic uptake after oral administration through the small intestine has been noted [51]. The increased activation of PBMCs noted here at 1 μg/mL could potentially be reached in serum after oral ingestion. A promising result of dietary intake of fucoidan in elderly Japanese men and women increased the immune response to seasonal influenza vaccination [52]. 

Fucoidan is not likely to be used as a sole therapy for cancer treatment, especially where there are known effective therapies. However, as a non-toxic edible natural product easily delivered orally or intranasally, fucoidan may find a role either to reduce side effects, or to enhance the therapeutic effects in combination with conventional therapies. 

## 4. Materials and Methods

### 4.1. Materials

Fucoidan extracts from *Fucus vesiculosus* (FV), *Undaria pinnatifida* (UP) and *Macrocystis pyrifera* (MP) were provided by Marinova Pty Ltd. (Cambridge, Australia). The specific properties of each extract are described in Table 1 and Table 2. Fucoidan puriy is calculated as the sum of neutral carbohydrates, sulfate, acetylation and cations, from the hydrolysed isolated polymer.

Prostate cancer cell line PC3 was purchased from ATCC and used in vitro according to ATCC culture specifications. The cell line was checked for its mycoplasma-free status before experiments. 

PBMCs were isolated from blood samples purchased from the French Blood Institute collection centre (Bordeaux, France) where they were collected from consented healthy human donors following collection guidelines and according to the ethics committee. Samples were used in this study according to the convention agreement (#18PLER018) with the French Blood Institute collection centre.

### 4.2. Proliferation and Activation of PBMCs

Briefly, PBMCs activated with or without αCD3 at either 0.25 or 0.5 μg/mL were plated in the presence and absence of each of the 3 fucoidan extracts (UP, FV, MP) and assessed at 4 concentrations of 0, 10, 50, and 100 µg/mL. Immune cell proliferation (by means of cell confluence quantification—as surrogate) was followed by live cell imaging. In addition, culture supernatants were retrieved 72 h post-plating and treatment, for the quantification of the IFNγ released levels, as a surrogate of immune cell activation. 

### 4.3. Data Acquisition and Analysis of PBMCs and IFNγ

Image acquisition started after immune cell seeding, when the test compound treatment was applied. Phase contrast images were acquired on an IncuCyte ZOOM™ Live cell imager using a 10× objective, with 1 image every 2 h during the 5-day monitoring period. Image analysis was performed using IncuCyte ZOOM™ software following application of a segmentation mask analysis on phase contrast images to identify cell surface. Data were then analyzed and plotted using Graph Pad Prism^®^ v6.01 software.

In addition, 72 h following culture initiation and treatments, supernatants were collected and effects of test compounds were evaluated on immune cell activation by means of the quantification of IFNγ released levels, as a key representative surrogate. IFNγ quantification was performed using a specific Homogenous Time-Resolved Fluorescence, HTRF-based detection kit and TECAN Infinite F500 microplate reader.

### 4.4. The Immune Cell-Mediated Tumor Killing Activity Assay on PC3 Prostate Tumor Cells

Prior to its use in the killing assay, the PC3 prostate tumor cell line was first transduced and modified in a clonal population expressing a nuclear red fluorescent label (using Nuclight Red lentivirus). After their modification, tumor cells were seeded and cultured for 24 h, and then co-cultured with human effector immune cells (PBMC) activated with anti-CD3 at either 0.05 or 0.5 μg/mL, in the presence and absence of anti-PD1 antibody (Nivolumab, 1 μg/mL), and incubated in the presence and absence of each of FV, UP and MP—assessed at 0, 0.25, 0.5, 1, 5, 10, 25 and 50 µg/mL. In addition, the effects of the fucoidans were directly evaluated on tumor cells (cultured alone), in dose-dependent responses at 0, 0.25, 0.5, 1, 5, 10, 25 and 50 µg/mL. Tumor cell proliferation and apoptosis were followed by live cell imaging (based on a nuclear fluorescence and apoptosis specific fluorescence probes). In addition, supernatants from immune/tumor cell co-cultures were retrieved 72 h after co-culture initiation and treatment for the quantification of the IFNγ released levels, as a surrogate of immune cell activation/activity.

### 4.5. Data Acquisition and Analysis of PC3 Cells

Image acquisition started 24 h after tumor cell seeding, when the test compound treatments were applied (at the tumor/immune cell co-culture initiation). Phase contrast, green channel (fluorescent caspase 3/7 apoptosis probe) and red channel (fluorescent tumor nuclear probe) images were acquired on an IncuCyte ZOOM™ Live cell imager using a 10× objective, with 1 image every 3–4 h during 5 days monitoring period. Image analyses were performed using IncuCyte ZOOM™ software following application of a segmentation mask analysis on phase contrast images to identify cell surface, on red fluorescence images to select tumor cells (expressing the red fluorescent nuclear probe) and on green fluorescence images to identify apoptotic cells (Caspase 3/7 probe; DEVD-NucView™488). Overlay segmentation analysis was applied to identify apoptotic tumor cells. Data were analyzed and plotted using Graph Pad Prism^®^ v6.01 software.

### 4.6. Statistical Analysis

All the data were statistically analyzed. Normality distribution was evaluated and appropriate statistical analysis test was applied. For the IFNg level quantification data, variance analysis was performed (two-way ANOVA) and the Tukey’s test for post-hoc analysis was applied. For kinetic monitoring data, AUC (Area Under Curve) was analyzed and Student’s *t*-test was used. *p* < 0.05 was considered as minimal level to be significant (GraphPad PRISM, La Jolla, CA, USA).

## 5. Conclusions

We demonstrated here that fucoidan extracts from three species of macroalgae enhanced the proliferation and IFNγ secretion of PBMCs. This enhancement was additive to the effects of the checkpoint inhibitor, Nivolumab and appeared independent of the fucoidan carbohydrate profile. The increased activity did not result in increased killing of PC3 cancer cells, but, instead, all fucoidan extracts had a direct inhibitory effect on the cancer cells. Fucoidan may have a novel role in the attenuation of cancer cells, whilst simultaneously enhancing immune activity of PBMCs.

## Figures and Tables

**Table 1:** Description of fucoidan extracts from *Fucus vesiculosus* (FV), *Undaria pinnatifida* (UP) and *Macrocysitis pyrifera* (MP).

Fucoidan Extract	Neutral Carbohydrates	Sulfate	Fucoidan	Polyphenol	Molecular Weight (kDa)
FV	62.7%	25.0%	92.9%	3.3%	49.6
UP	43.5%	25.9%	86.0%	<2%	46.8
MP	47.0%	25.7%	80.0%	<2%	66.0

**Table 2:** Weight percent of fucoidan sugars.

	Fucose	Xylose	Galactose	Arabinose	Rhamnose
FV	46%	7%	4%	1%	0%
UP	21%	1%	18%	1%	0%
MP	31%	1%	7%	1%	1%

## Data Availability

The data represented in this study are available on request from the corresponding author. The data are not publicly available due to proprietary nature.

## Supplementary Materials

The following are available online at https://www.mdpi.com/article/10.3390/md20010012/s1, Figure S1: Real-time live cell monitoring of inactivated PBMCs, under treatment with fucoidans. Figure S2: Images of cell proliferation and clusterization of activated PBMCs in the presence of αCD3 (0.25 μg/mL) and fucoidans (0–100 μg/mL). Figure S3: Images of cell proliferation and clusterization of activated PBMCs in the presence of αCD3 (0.5 μg/mL) and fucoidans (0–100 μg/mL). Figure S4: Changes in cell confluence (%) of PBMCs in the presence of fucoidans. Figure S5: Real-time live cell monitoring of inactivated and αCD3-activated PBMCs in the presence and absence of Nivolumab. Figure S6: Real-time live cell monitoring of prostate PC3 tumor cell killing mediated by activated PBMCs, under treatment with fucoidans. Figure S7: Real-time live cell monitoring of prostate PC3 tumor cell killing mediated by activated PBMCs in the presence of αCD3 (0.5 µg/mL), under treatment with fucoidans.
