# Supplementary material for: Fucoidan Independently Enhances Activity in Human Immune Cells and Has a Cytostatic Effect on Prostate Cancer Cells in the Presence of Nivolumab
Source: Mar Drugs. 2021 Dec 22;20(1):12. doi: 10.3390/md20010012 (PMC8779234; doi:10.3390/md20010012)
Supplement: Supplementary file 1 [file marinedrugs-20-00012-s001.zip › marinedrugs-1471645-supplementary.pdf]

Supplementary Information
Fucoidan Independently Enhances Activity in Human Immune Cells and Has a Cytostatic Effect on Prostate Cancer Cells in the Presence of Nivolumab

Ah Young Park [1,*], Imane Nafia [2], Damien N. Stringer [1], Samuel S. Karpiniec [1] and J. Helen Fitton [1,3]

1 Marinova Pty Ltd., Cambridge TAS7170, Australia; damien.stringer@marinova.com.au (D.N.S); sam.karpiniec@marinova.com.au (S.S.K.); drfitton@rdadvisor.com (J.H.F.)
2 Explicyte Immuno-Oncology, 33000 Bordeaux, France; i.nafia@explicyte.com
3 RDadvisor, Hobart TAS7006, Australia
* Correspondence: ahyoung.park@marinova.com.au; Tel.: +61-3-6248-5800

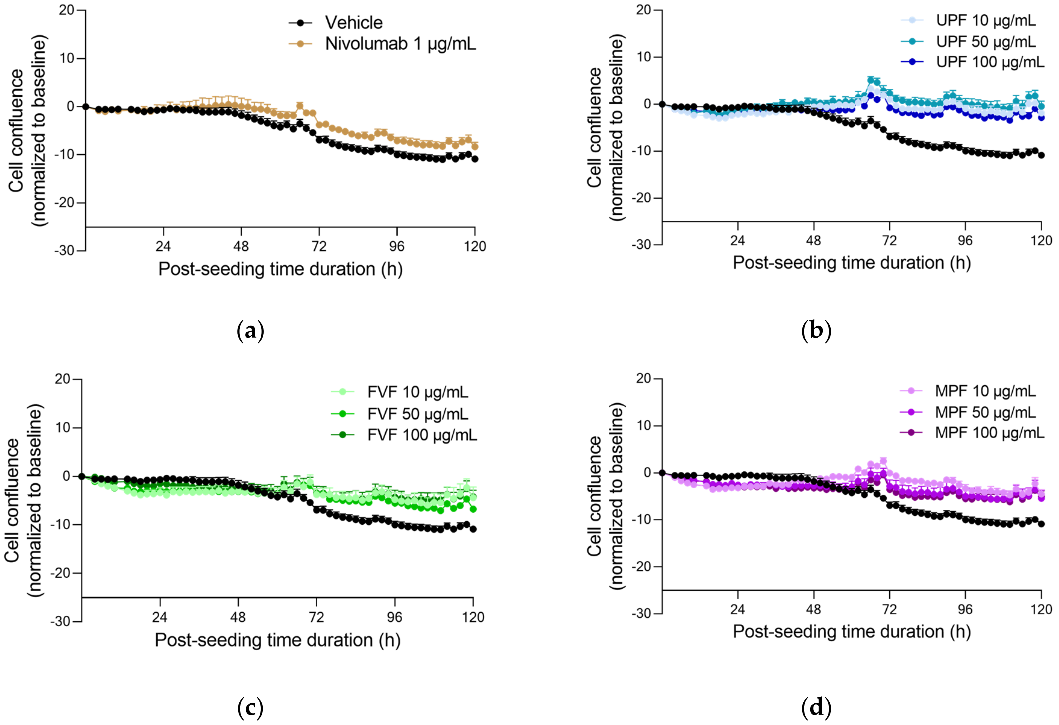

Figure S1. Real-time live cell monitoring of inactivated PBMCs, under treatment with fucoidans. PBMCs were seeded in the absence of αCD3, and treated with increasing doses of fucoidans (**a**) Nivolumab, as positive control (**b**) UP (**c**) FV and (**d**) MPF. Cell confluence was monitored and quantified – as surrogate of cell proliferation, over a period of ~5 days. Data were normalized and corrected to the baseline and are expressed as means ± SEM.

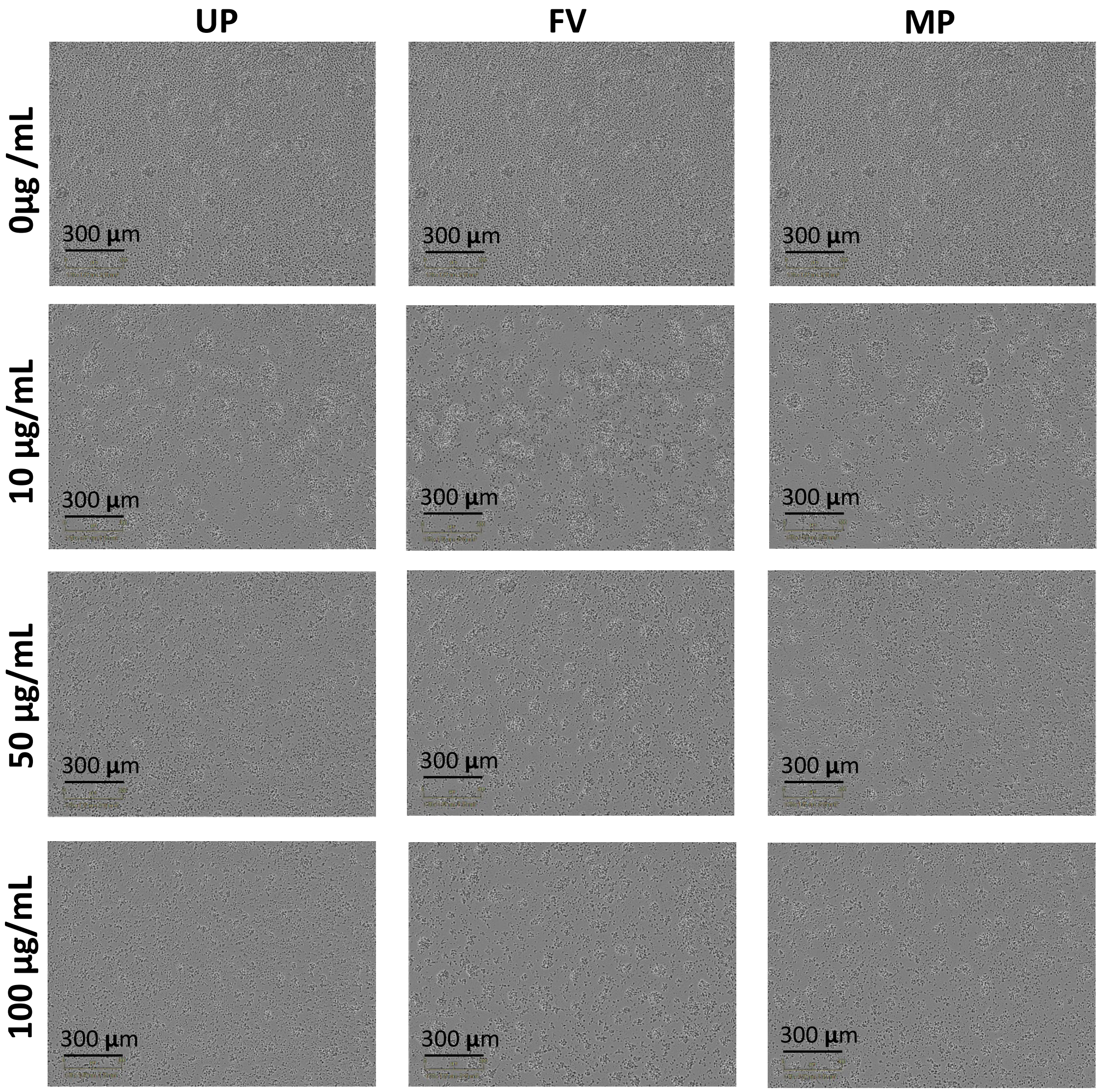

Figure S2. Images of cell proliferation and clusterization of activated PBMCs in the presence of αCD3 (0.25 µg/mL) and fucoidans (0–100 µg/mL).

PBMCs were seeded in the presence of αCD3 (0.25 µg/mL), and treated with increasing doses of fucoidans. Representative image fields of cell proliferation and clusterization induced upon 96 h treatment with test fucoidan extracts.

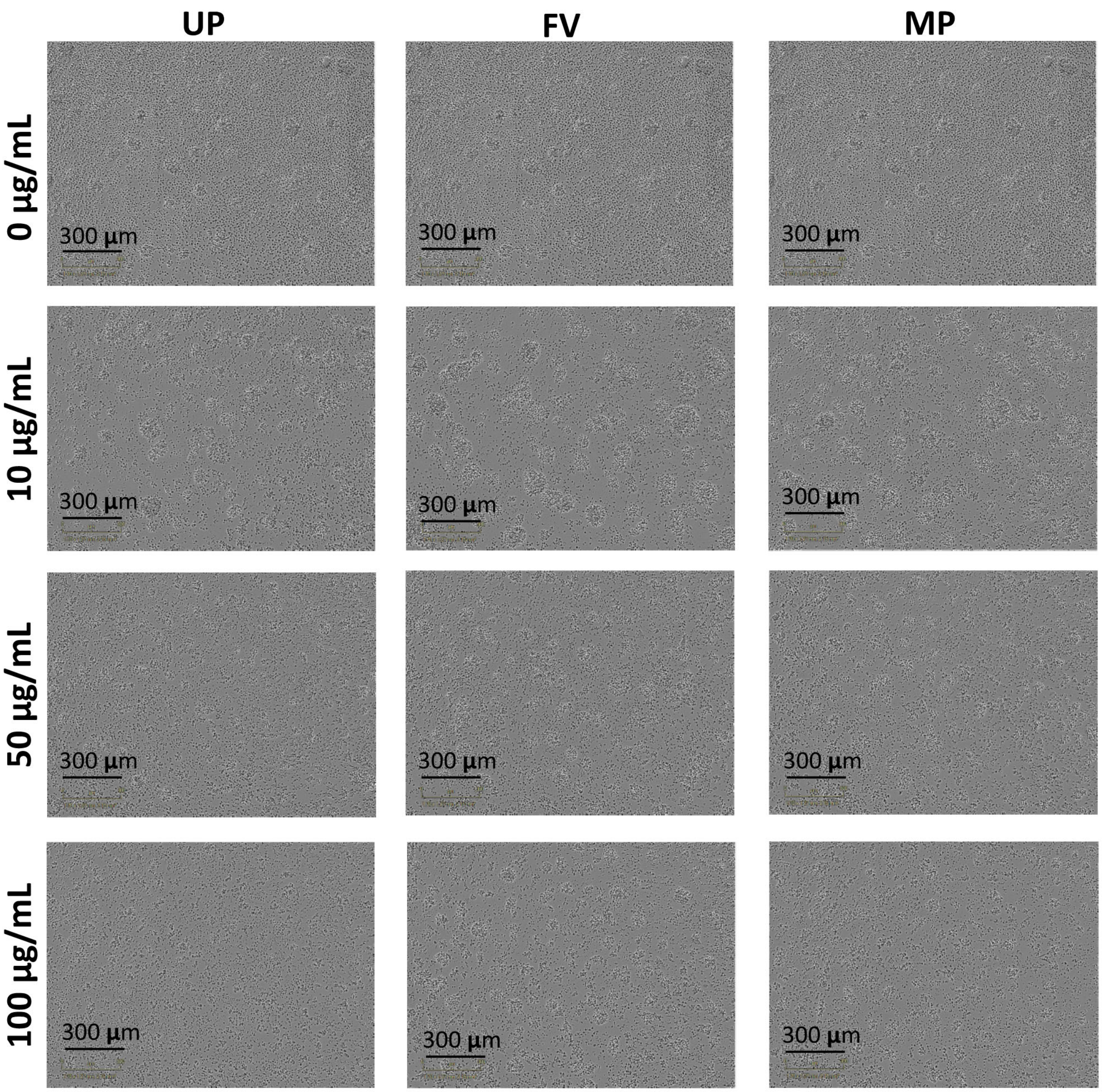

Figure S3. Images of cell proliferation and clusterization of activated PBMCs in the presence of αCD3 (0.5 μg/mL) and fucoidans (0–100 μg/mL).

PBMCs were seeded in the presence of αCD3 (0.5 μg/mL), and treated with increasing doses of test compounds. Representative image fields of cell proliferation and clusterization induced upon 96 h treatment with test fucoidan extracts.

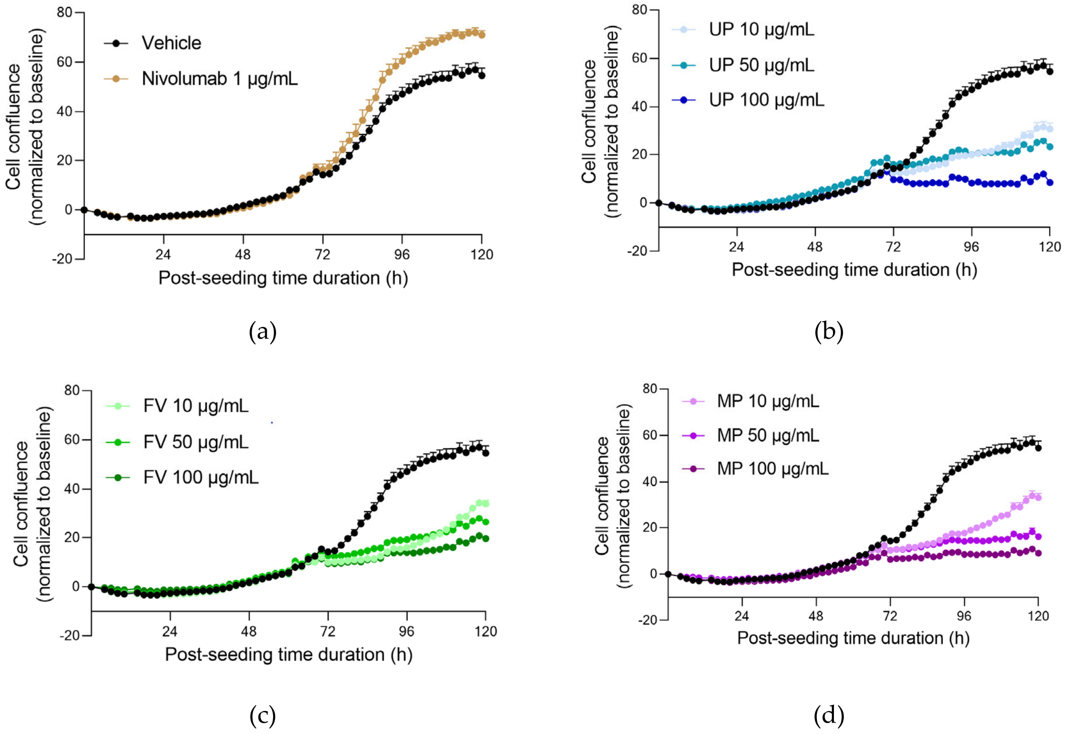

Figure S4. Changes in cell confluence of PBMCs in the presence of fucoidans.
PBMCs were seeded in the presence of αCD3 at 0.25 µg/mL and treated with increasing dose of (**a**) Nivolumab as positive control, (**b**) UP, (**c**) FV and (**d**) MP. Cell confluence was monitored and quantified – as surrogate of cell proliferation over a period of ~5 days. Data were normalised and corrected to the baseline and are expressed as means ± SEM.

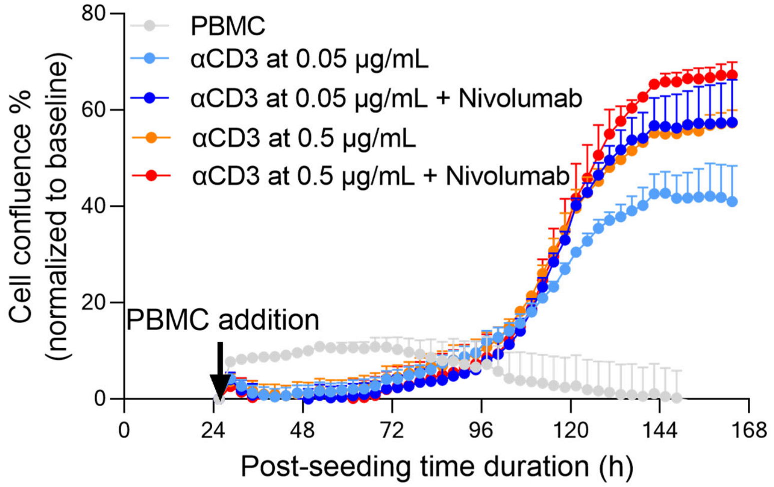

Figure S5. Real-time live cell monitoring of inactivated and αCD3-activated PBMCs in the presence and absence of Nivolumab. PBMCs were seeded either in the absence or the presence of αCD3, with and without Nivolumab at 1 µg/mL. Cell confluence was monitored and quantified – as surrogate of cell proliferation, over a period of ~5 days. Data were normalized and corrected to the baseline and are expressed as means ± SEM.

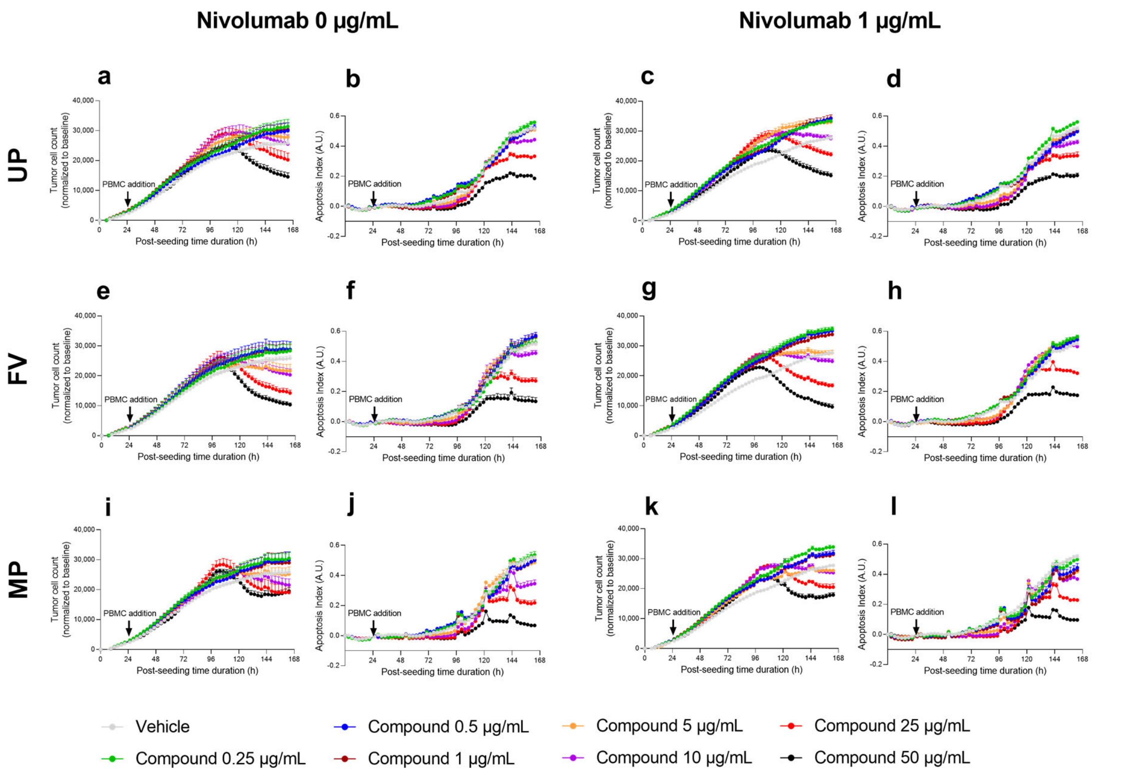

Figure S6. Real-time live cell monitoring of prostate PC3 tumor cell killing mediated by activated PBMCs, under treatment with fucoidans. Prostate PC3 tumor cells were seeded and 24h later were cocultured with PBMCs, in the presence of αCD3 (0.05 µg/mL), and in the presence and absence of Nivolumab (1 µg/mL), and treated with increasing doses of each of test compounds – UP (**a**–**d**), FV (**e**–**h**), and MP (**i**–**l**) under the different conditions of treatments with compounds. Tumor cell count (**a**, **e**, **i**, **c**, **g**, **k**) and apoptosis (**b**, **f**, **j**, **d**, **h**, **l**) were monitored and quantified over a period of ~5 days, by mean of a NucRed probe expression and caspase 3/7 fluorescent probe, respectively, as surrogate measures of immune cell killing activity towards tumor cells. Apoptosis is represented as an index evaluated with respect to the apoptosis events and cell number in each condition. Data were normalized and corrected to the baseline and are expressed as means ± SEM.

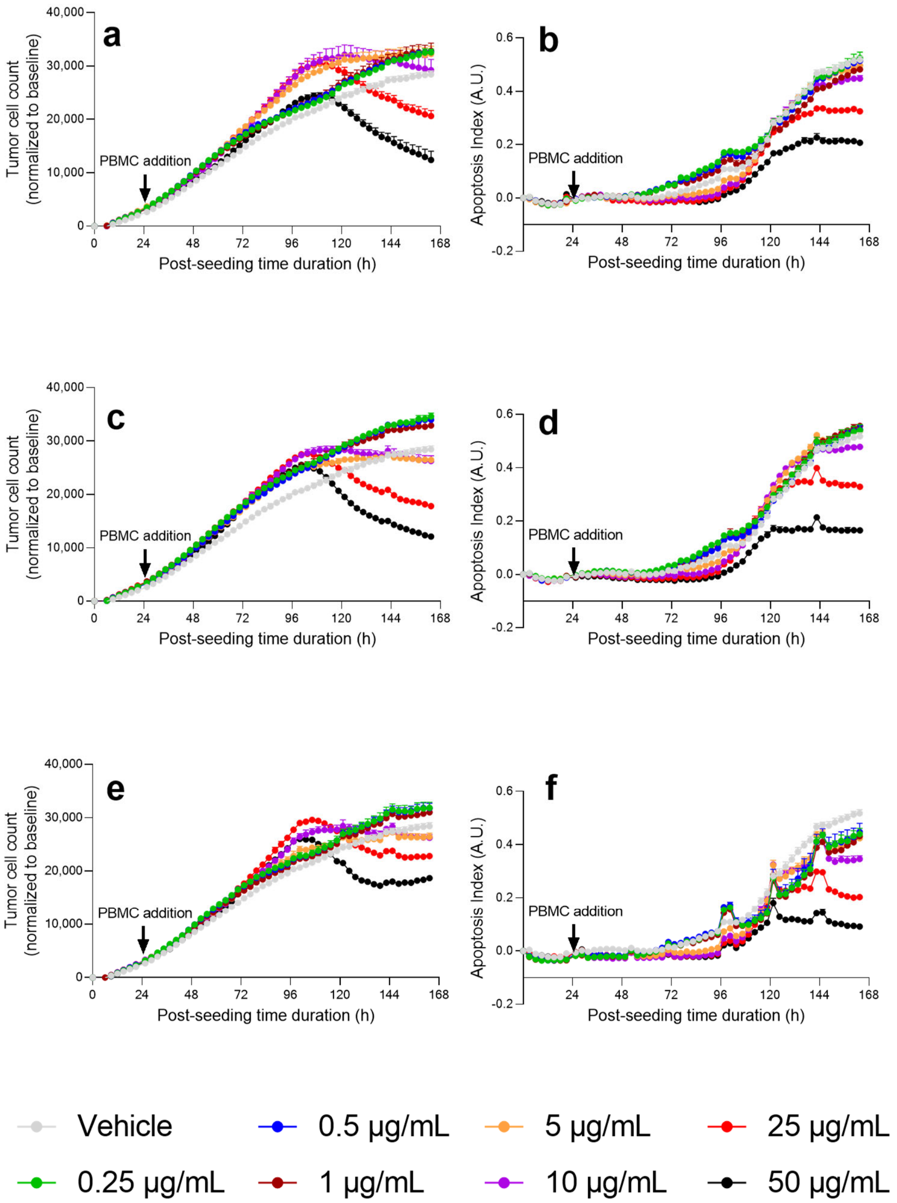

Figure S7: Real-time live cell monitoring of prostate PC3 tumor cell killing mediated by activated PBMCs in the presence of αCD3 (0.5 µg/mL), under treatment with fucoidans. Prostate PC3 tumor cells were seeded and 24h later were cocultured with PBMCs, in the presence of αCD3 (0.5 µg/mL), and in the absence of Nivolumab, and treated with increasing doses of each of test compounds – UP (**a**–**b**), FV (**c**–**d**), and MP (**e**–**f**) under the different conditions of treatments with compounds. Tumor cell count (**a**, **c**, **e**) and apoptosis (**b**, **d**, **f**) were monitored and quantified over a period of ~5 days, by mean of a NucRed probe expression and caspase 3/7 fluorescent probe, respectively, as surrogate measures of immune cell killing activity towards tumor cells. Apoptosis is represented as an index evaluated with respect to the apoptosis events and cell number in each condition. Data were normalized and corrected to the baseline and are expressed as means ± SEM.
